# Macaque parvocellular mediodorsal thalamus: dissociable contributions to learning and adaptive decision‐making

**DOI:** 10.1111/ejn.14078

**Published:** 2018-08-16

**Authors:** Subhojit Chakraborty, Zakaria Ouhaz, Stuart Mason, Anna S. Mitchell

**Affiliations:** ^1^ Department of Experimental Psychology University of Oxford The Tinsley Building, Mansfield Road Oxford OX1 3SR UK

**Keywords:** episodic memory, frontal‐temporal cortex network, medial prefrontal cortex network

## Abstract

Distributed brain networks govern adaptive decision‐making, new learning and rapid updating of information. However, the functional contribution of the rhesus macaque monkey parvocellular nucleus of the mediodorsal thalamus (MDpc) in these key higher cognitive processes remains unknown. This study investigated the impact of MDpc damage in cognition. Preoperatively, animals were trained on an object‐in‐place scene discrimination task that assesses rapid learning of novel information within each session. Bilateral neurotoxic (NMDA and ibotenic acid) MDpc lesions did not impair new learning unless the monkey had also sustained damage to the magnocellular division of the MD (MDmc). Contralateral unilateral MDpc and MDmc damage also impaired new learning, while selective unilateral MDmc damage produced new learning deficits that eventually resolved with repeated testing. In contrast, during food reward (satiety) devaluation, monkeys with either bilateral MDpc damage or combined MDpc and MDmc damage showed attenuated food reward preferences compared to unoperated control monkeys; the selective unilateral MDmc damage left performance intact. Our preliminary results demonstrate selective dissociable roles for the two adjacent nuclei of the primate MD, namely, MDpc, as part of a frontal cortical network, and the MDmc, as part of a frontal‐temporal cortical network, in learning, memory and the cognitive control of behavioural choices after changes in reward value. Moreover, the functional cognitive deficits produced after differing MD damage show that the different subdivisions of the MD thalamus support distributed neural networks to rapidly and fluidly incorporate task‐relevant information, in order to optimise the animals’ ability to receive rewards.

Mammalian mediodorsal thalamus contributes to higher cognitive functions, including new learning, rapid updating and adaptive decision‐making. Current animal evidence demonstrates that this occurs through interactions across distributed cortical and subcortical neural networks (Parnaudeau *et al*., 2013, 2015; Browning *et al*., 2015; Schmitt *et al*., 2017). Thus far, this causal evidence has focused on neurotoxic lesions to the magnocellular subdivision of the MD in monkeys (MDmc); (Mitchell *et al*., 2007a,b; Mitchell & Gaffan, 2008; Izquierdo & Murray, 2010; Mitchell & Chakraborty, 2013; Browning *et al*., 2015; Mitchell, 2015) or, in mice, combined optogenetics and electrophysiology recordings of either the whole of the MD (Parnaudeau *et al*., 2013, 2015; Bolkan *et al*., 2017) or the lateral MD subdivision (Schmitt *et al*., 2017). Work in the Mitchell laboratory over the past decade has established the importance of an intact MDmc in cognitive tasks that require the monkeys to rapidly process trial‐relevant task information when involved in new learning or adaptive decision‐making (Mitchell *et al*., 2007a,b, 2008, 2014; Browning *et al*., 2015; Chakraborty *et al*., 2016). However, monkeys with MDmc damage showed intact performance when required to retrieve or recognise preoperatively learned task‐relevant information across different cognitive tasks (Mitchell *et al*., 2007a, 2008; Mitchell & Gaffan, 2008), supporting the notion that an intact MDmc is, therefore, important for updating rather than the active maintenance of the old information *per se* (Mitchell, 2015). However, the MD is diverse with its different subdivisions uniquely interconnected to interdependent cortical and subcortical brain structures. So to best advance our understanding about how the MD contributes to higher cognitive functions, it is critical to investigate each of its different subdivisions; this study focused on the specific contribution of the parvocellular subdivision (MDpc). Thus, an outstanding question remains about the effects on learning, memory and adaptive decision‐making after MDpc damage in non‐human primates.

In primates, the MDpc subdivision is situated in the central part of the mediodorsal thalamus, with the MDmc on its more medial boundary and the lateral MD on its lateral boundary. Neuroanatomical tracing studies in non‐human primates show that the MDpc is reciprocally and densely connected to the dorsolateral prefrontal cortex (DLPFC: areas 9 and 46), frontopolar cortex (area 10), orbitofrontal cortex (OFC: areas 12 and 13) and the dorsal anterior cingulate cortex (areas 24, 32 and 14). It also receives non‐reciprocal inputs from several structures including: the ventrolateral prefrontal cortex and the piriform cortex (Preuss & Goldman‐Rakic, 1987; Russchen *et al*., 1987; Barbas *et al*., 1991; Dermon & Barbas, 1994; Bachevalier *et al*., 1997; Cavada *et al*., 2000; Haber & McFarland, 2001; Xiao *et al*., 2009; Garcia‐Cabezas & Barbas, 2017). The identification of dual cortico‐thalamic connections that originate from layer 5 (driver), and from layer 6 (modulator) of the DLPFC and terminate in the MDpc (Schwartz *et al*., 1991), helped inspire Guillery and colleagues to propose an alternative categorisation of the thalamic nuclei, and an alternative theory about cortico‐cortical communication, one that involves ongoing interactions between the thalamus and the cortex, via transthalamic routes of communication (Guillery, 1995, 2017; Sherman & Guillery, 2006, 2013). The MDpc does not receive direct sensory input (driver signals) from the periphery as the primary thalamic relays (e.g. the lateral geniculate nucleus) do; instead its driver signal is proposed to originate from the layer 5 cortico‐thalamic pathways, thus the MDpc has been described as a ‘higher order thalamic relay’ (Guillery, 1995, 2017; Sherman & Guillery, 2006, 2013; Mitchell, 2015). In addition to the dense cortical connections, the MDpc is also a node within the dorsal cortico‐striato‐thalamic loop. Specifically, MDpc is integrated into this dorsal loop receiving GABAergic inputs from the dorsal caudate–putamen via the internal segment of the globus pallidus (Alexander *et al*., 1986; Haber & McFarland, 2001; Haber & Calzavara, 2009; Haber & Knutson, 2010). The MDpc also receives neuromodulatory inputs from other parts of the midbrain, brainstem and reticular thalamus (Jones, 2007). Single relay neuron filled tracer studies have demonstrated that in rats, the central part of the MD, which may be considered similar to the primate MDpc (Mitchell & Chakraborty, 2013), sends axons to widespread frontal cortical layers and areas (Kuramoto *et al*., 2017), suggesting the ability of this thalamic relay to influence many distributed frontal cortical functions. Thus, this neural connectivity suggests that the MDpc should contribute to supporting several cognitive functions governed by the frontal cortex, and that this functioning is modulated via links with layer 6 of the cortex, the dorsal cortico‐striatal feedback loop, and the midbrain and brainstem.

Given that stroke patients with damage to the MD typically have variable arterial perfusion and differing amounts of neuropathology both in the white matter of the diencephalon and in several nuclei of the medial, dorsal and limbic thalamus, including the anterior thalamus, MD and the intralaminar nuclei (Van der Werf *et al*., 2000, 2003; Schmahmann, 2003), for now, it remains vital to tease apart the distinct involvement of the different subdivisions of the MD in higher cognitive functions with targeted surgical interventions in animal models. Thus, the present experiment tested for the first time how rhesus macaques with differing loci of neurotoxic lesion damage to the MDpc contribute to two different learning tasks and a food reward devaluation task. Specially, we assessed the contribution of the MDpc in rapid within‐session new learning of object‐in‐place scene discriminations (Gaffan, 1994; Mitchell *et al*., 2007a, 2007b), as well as slower across session learning of object–reward associations to a 90% criterion (Mitchell *et al*., 2007b), and adaptive decision‐making processes, using a reward‐based reinforcer devaluation paradigm (Málková *et al*., 1997; Baxter *et al*., 2000; Izquierdo & Murray, 2004; Mitchell *et al*., 2007b). Given the distinctive interdependent connectivity of the MDpc and MDmc (Mitchell & Chakraborty, 2013; Mitchell, 2015; Ouhaz *et al*., 2018), we deemed it important to compare whether the performance of monkeys after neurotoxic MDpc lesions is similar to, or dissociable from, that of monkeys with neurotoxic MDmc lesions. Thus, the results from this study are also compared with previously published work in non‐human primates from our laboratory using the same object‐in‐place scene discrimination learning task (Mitchell *et al*., 2007a; Browning *et al*., 2015) and the same reward devaluation task (Mitchell *et al*., 2007b) after either bilateral or unilateral neurotoxic lesions to MDmc.

## Materials and methods

### Subjects

In this study, there were 8 (two females) rhesus macaque monkeys (*Macaca mulatta*) aged between 4 and 6 years at the beginning of behavioural training. One male monkey (MDP1) received selective bilateral MDpc lesions; male monkey (MDP2) received selective unilateral MDpc lesions in one hemisphere combined with selective unilateral MDmc lesions in the contralateral hemisphere; male monkey (MDP3) received unilateral MDmc lesions that extended into the midline thalamus and male monkey (MDP4) received combined bilateral MDpc and MDmc lesions in both hemispheres. The performance of these four monkeys was compared to four unoperated control monkeys who experienced the exact same testing conditions, tasks, stimuli and order of presentation of experiments, whose data have been previously published (Mitchell *et al*., 2007a,b).

All experimental procedures were performed in compliance with the United Kingdom Animals (Scientific Procedures) Act of 1986. A Home Office (UK) Project License obtained after review by the University of Oxford Animal Care and Ethical Review committee licensed all procedures. The animals were socially housed together in same‐sex groups of between 2 and 6 animals. The housing and husbandry were in compliance with the ARRIVE guidelines of the European Directive (2010/63/EU) for the care and use of laboratory animals.

### Apparatus

The computer‐controlled test apparatus was identical to that previously described (Mitchell *et al*., 2007a,b). Briefly, monkeys sat in a transport box fixed to the front of a large touch‐sensitive colour monitor that displayed the visual stimuli for all the experiments. Monkeys reached out through the bars of the transport box to respond on the touch screen and collect their food reward pellets from a hopper that were automatically dispensed by the computer. Monkeys were monitored remotely via closed circuit cameras and display monitors throughout the testing period.

### Procedures

**Figure 1:**
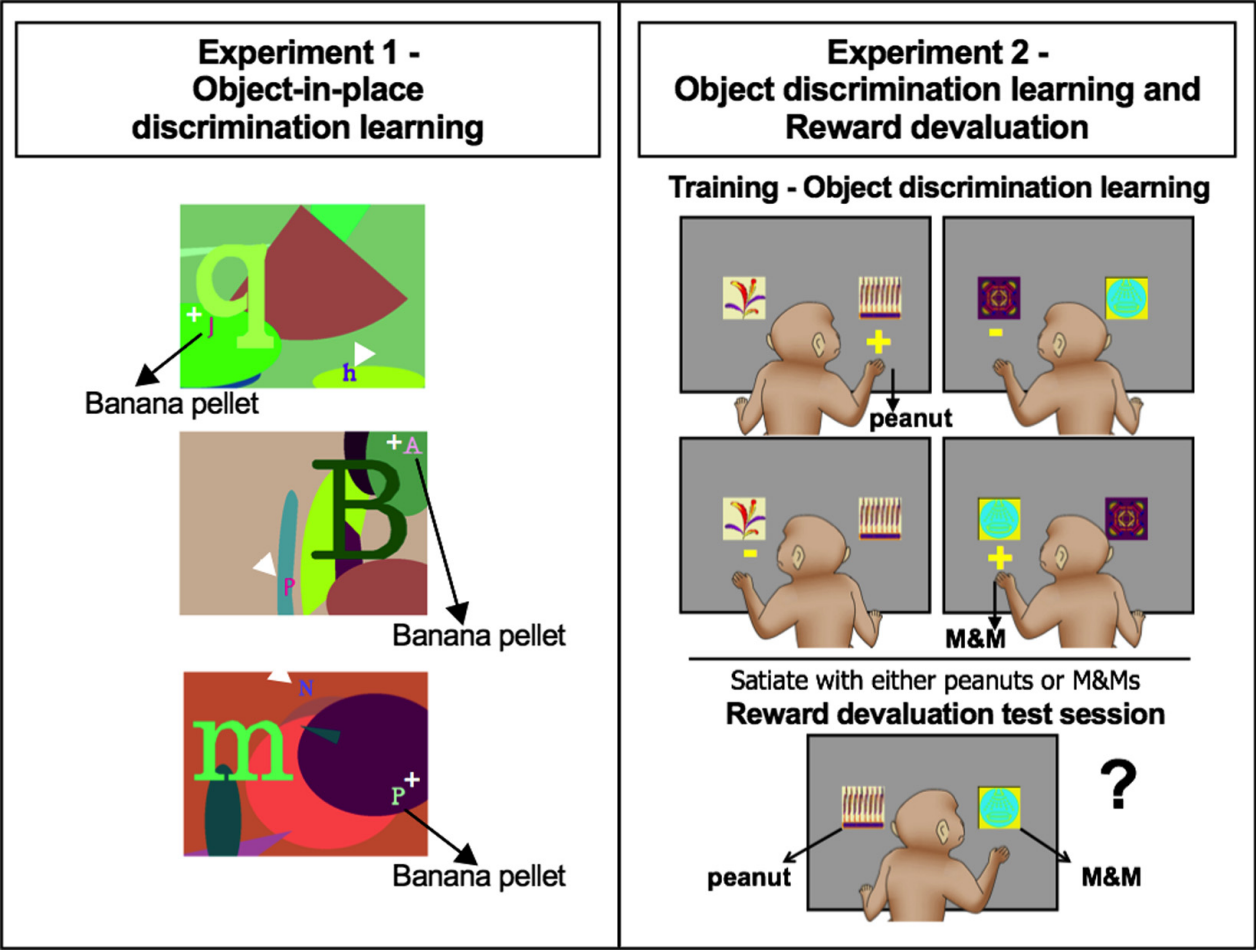
Experimental design. Left: Three examples of object‐in‐place scene discrimination stimuli ‘scenes’ used in Experiment 1 in this study. Monkeys respond to each ‘scene’ by touching one of the two foreground objects. One of the two foreground typographic objects in each ‘scene’ denoted by ‘S+’ is arbitrarily designated as correct (reward). The ‘S−’ indicates the locations of the unrewarded foreground objects in each ‘scene.’ The locations and identities of the foreground objects are fixed within each scene but vary across scenes. Right: object–reward association learning and food devaluation paradigm. During training (object–reward association learning), monkeys are presented with two clipart images per trial (60 pairs) and learn which object rewards them with a peanut or an M&M or no reward. After reaching criterion, food reward devaluation is conducted. Monkeys are satiated with one food reward just prior to the test session. During devaluation test sessions, pairs of rewarded objects only are presented (30 pairs in total) and the monkey chooses between the two objects to receive either a peanut or an M&M reward. Adapted from Browning *et al*. (2015).

Figure 1 provides a visual model of the two experiments incorporating the three cognitive tasks and order of testing for the monkeys.

### Experiment 1: object‐in‐place scene discrimination learning

This object‐in‐place scene discrimination learning task was adapted (Gaffan, 1994). Briefly, each trial consisted of an artificially constructed ‘scene.’ There were two foreground ‘objects’ for each discrimination, one correct (rewarded) and the other incorrect (non‐rewarded), consisting of randomly selected small‐coloured typographic characters each placed in a constant location. Each discrimination scene was unique in that they varied in several randomly selected attributes, including (1) the background colour of the screen, (2) the location of ellipses on the screen, (3) the colour, size and orientation of ellipse segments, (4) the typographic character, clearly distinct in size from the foreground objects and (5) the colour of the typographic character. All the colours were assigned with the constraint that the foreground objects should be visible (i.e. there was a minimum separation in colour space between the colours of a foreground object and the colour of any element of its local background).

#### Behavioural training

Pretraining followed a previously published protocol (Mitchell *et al*., 2007a,b). The behavioural training began once the monkeys were reliably touching the foreground objects when presented with a new scene and completing 50 trials a day with minimal accuracy errors (i.e. touching any location on the screen other than the foreground typographic characters). Problems were introduced with two foreground objects (one correct and one incorrect, as described earlier), and the number of scenes given in each session was gradually increased, based on each monkey's performance. The locations and identities of the foreground objects were fixed within each scene but varied between scenes. As these scenes were randomly generated, an infinite number of unique scenes could be presented. A touch to the correct object caused the object to flash for 2 s, whereas the incorrect object stayed on screen along with the colourful visual background scene, then the screen blanked and a reward pellet was delivered into the hopper. A touch to the incorrect object caused the screen to blank immediately, no reward was given, and an ITI of 10 s followed before the start of the next trial. For the first presentation of the list of novel scenes only, incorrect responses were followed by a correction trial in which the scene was re‐presented with only the correct object present. Touches anywhere else in the scene caused the screen to blank, and the trial was repeated.

In the final version of the task, the monkey was required to learn a novel set of 20 new object‐in‐place scene discriminations within each testing session (or 10 novel scenes for MDP2 and MDP3 as these two monkeys could not maintain stable performance while learning 20 new scenes each day), by being exposed to an initial run through the set of 20 (or 10 for MDP2 and MDP3) discriminations followed by seven repetition trials of the set of 20 (or 10 for MDP2 and MDP3) discriminations within the session (in the same order in each of the repetitions through the set of discriminations). On the next daily testing session, a novel set of 20 (or 10 for MDP2 and MDP3) discriminations was presented and learnt within the session in the same fashion as mentioned above, and so on. During daily learning, performance on the first presentation of the discriminations (Trial 1) is at chance, as the monkey has no information about which of the two foreground objects is the correct object to choose on the very first exposure of each discrimination. Then, through subsequent repetitions of the same discriminations within the session (Trials 2–8), the monkey learns the discriminations rapidly. Once stable learning is established within each session across several weeks of testing with new discrimination problems presented in each testing session, a monkey has a rest period of 2 weeks (equivalent in duration to a ‘postoperative rest’) then a preoperative performance test for 13 days is conducted. For Days 1 and 2 of the performance test, the monkey receives 1 session of 10 (or 5 for MDP2 and MDP3) novel object‐in‐place scene discriminations (with seven repetition trials within the session), again with novel discriminations used each day. Then, for Days 3–13, the monkey receives their preoperative performance test with 20 (or 10 for MDP2 and MDP3) novel discriminations each day and seven repetition trials within each session. The preoperative performance test data were analysed from the sessions completed on Days 4–13. After surgery and 2 weeks postoperative rest, an identical method for the postoperative performance test was followed to obtain postoperative within‐session learning data from the last 10 postoperative sessions (Days 4–13). Proficiency in preoperative (Pre) and postoperative (Posttest1) within‐session learning in this task is expressed as average percentage errors in repetition trials 2–8 across the final 10 sessions of testing (i.e. Days 4–13). After the first postoperative performance test was complete, all monkeys then started training and testing for Experiment 2: Object–reward association learning and devaluation. After completing Experiment 2, all monkeys completed another postoperative test (Posttest2) of the object‐in‐place scene discrimination task, as per Posttest1.

### Experiment 2: object discrimination learning and reinforcer devaluation

#### Object discrimination learning

The automated task and stimuli were identical to the previously published procedure (Mitchell *et al*., 2007b). After the first postoperative performance test (Posttest1) was complete, all monkeys started learning the same set of distinct pairs of clipart objects as two‐choice object discrimination learning problems with each pair representing one object discrimination problem (60 problems in total). Each trial began with the presentation of one pair of clipart images against a gray background, one on the left side of the screen and one on the right, and these positions were pseudo‐randomised across trials. One object was arbitrarily assigned correct in each pair. Touching the correct object caused it to flash for 2 s (the incorrect object immediately disappeared), and it also resulted in the immediate delivery of a reward of either a half‐peanut or an M&M chocolate candy into the food hopper. Half of the rewarded objects delivered a half‐peanut, and the other rewarded objects resulted in an M&M. Each problem appeared once in each session. Touches to the incorrect object caused both objects to disappear immediately, and no reward was delivered. The ITI was 30 s after a choice was made, and a touch to the screen during the ITI reset it. Training continued until reaching a criterion of 270 or more correct responses over five consecutive sessions (90% or greater correct).

#### Reward devaluation

On reaching criterion, a series of sessions of critical trials were presented in which the 60 rewarded objects were randomly assigned to create 30 pairs of critical trials, each pair of trials offering a choice between a peanut‐rewarded object and an M&M‐rewarded object (i.e. there were no non‐rewarded objects presented in the critical trials sessions). Some sessions of critical trials were preceded by a devaluation procedure in which the monkey was allowed to consume 1 of the 2 food rewards to satiation before beginning the critical trial session. The sequence of critical trial sessions was baseline, peanut devaluation, baseline and M&M devaluation (Test 1). The same sequence was repeated once (Test 2). Each critical trial session was separated by at least 1 standard training session (as mentioned above), and monkeys had at least 2 days of rest after a reward devaluation critical trial session. For the reward devaluation critical trial sessions, the monkey sat in their transport box in a separate, familiar room, and a plastic box was fixed to the front of the cage containing a known amount of food reinforcer (either M&Ms or peanuts). The monkey was left undisturbed for 15 min to consume this food. If the food was completely eaten, the box was refilled. The monkey was then observed closely, and once it had not taken any food for 5 min, the box was removed from the cage. Then, when the monkey's cheek pouches were not visibly full with food, it was moved to the testing cubicle and the critical food devaluation trial session begun. It is important to note that, during these critical devaluation trial sessions when the peanuts or M&Ms have been satiated, no further learning of object–reward associations can occur because each pair of objects followed by a food reward is only presented once during the critical devaluation session. The critical measure of performance was a score composed of the difference in the number of choices of objects paired with a particular food on critical baseline sessions and on critical devaluation sessions preceded by the selective satiation of that food being devalued. These scores were added together for each devalued food. This was calculated separately for each sequence of critical trial sessions (Test 1 and Test 2), and the mean was taken as the overall score. Higher positive difference scores indicate sensitivity to reinforcer devaluation.

#### Surgery

All monkeys had one neurosurgery. Neurosurgical procedures were performed in a dedicated operating theatre under aseptic conditions and aided by an operating microscope. Steroids (methylprednisolone, 20 mg/kg) were given the night before surgery intramuscularly (i.m.), and 4 doses were given 4–6 h apart [intravenously (i.v.) or i.m.] on the day of surgery to protect against intraoperative oedema and postoperative inflammation. Each monkey was sedated on the morning of surgery with a cocktail of ketamine (10 mg/kg) and xylazine (0.25–0.5 mg/kg, i.m.). Once sedated, the monkey was given atropine (0.05 mg/kg, i.m.) to reduce secretion, antibiotic (amoxicillin, 8.75 mg/kg) as prophylaxis against infection, opioid (buprenorphine 0.01 mg/kg, repeated twice at 4–6 h intervals on the day of surgery, i.v. or i.m.) and non‐steroidal anti‐inflammatory (meloxicam, 0.2 mg/kg, i.v.) agents for analgesia, and an H2 receptor antagonist (ranitidine, 1 mg/kg, i.v.) to protect against gastric ulceration as a side effect of the combination of steroid and non‐steroidal anti‐inflammatory treatment. The head was shaved and an intravenous cannula put in place for intraoperative delivery of fluids (warmed sterile saline drip, 5 mL/h/kg). The monkey was moved into the operating theatre, intubated, placed on sevoflurane anaesthesia (2.25–4%, to effect, in 100% oxygen) and then mechanically ventilated. A hot air blower (Bair Hugger) allowed maintenance of normal body temperature during surgery. Heart rate, oxygen saturation of haemoglobin, mean arterial blood pressure, tidal CO_2_, body temperature and respiration rate were monitored continuously throughout the surgery.

The monkey was then placed in a stereotaxic head holder and the head cleaned with alternating antimicrobial scrub and alcohol and draped to allow a midline incision. After opening the skin and underlying galea in layers, a large D‐shaped bone flap was created in the cranium over the area of the operation and the dura over the posterior part of the hemisphere was cut and retracted to the midline. Veins draining into the sagittal sinus were cauterised and cut. The hemisphere was retracted with a brain spoon, and the splenium of the corpus callosum (5 mm) was cut in the midline with a glass aspirator. The tela choroidea was cauterised at the midline, posterior and dorsal to the thalamus using a metal aspirator that was insulated to the tip. The posterior commissure, the third ventricle posterior to the thalamus and the most posterior 5 mm of the midline thalamus were exposed. Stereotaxic coordinates were set from the posterior commissure at the midline using the third ventricle as a guide by positioning a stereotaxic manipulator holding a sharp tipped 26‐gauge needle of a 10‐μL Hamilton syringe above this site. The monkey brain atlas (Ilinsky & Kultas‐Ilinsky, 1987) was used to calculate the coordinates of the intended lesion site.

For the MDpc lesions, the position of the mediodorsal thalamus was calculated to be +4.8 mm dorsal to the posterior commissure. The needle was then inserted into the mediodorsal thalamus with the first set of coordinates: anteroposterior (AP), +4.3 mm anterior to the posterior commissure; mediolateral (ML), ±2.2 mm lateral to the third ventricle; dorsoventral (DV), −3.0 mm from the calculated DV range of the mediodorsal thalamus (see above). The syringe initially was inserted more ventral to the intended lesion site (to −6.0 mm from this DV), was left to ‘settle’ in the brain for 3 mins and then drawn up to the intended DV to start the neurotoxic injection. For the second coordinates: AP, +4.3 mm; ML, ±2.2 mm; DV, −2.0 mm. The third, fourth and fifth pairs of coordinates were AP, +3.7 mm; ML, ±2.8 mm and DV, −3.0 mm; AP, +3.7 mm, ML, ±2.8 mm and DV, −2.0 mm; and AP, +3.0 mm, ML, ±3.3 mm and DV, −2.0 mm respectively. For all of these lesion sites, the needle passed through the cortex and corpus callosum before entering the mediodorsal thalamus.

For the MDmc lesions, the same reference coordinates were taken from the posterior commissure and third ventricle as described above. The DV for the dorsal thalamus was measured at the lesion site by visual observation of the needle tip resting directly on the thalamus. The needle was positioned for the first set of coordinates: AP, +4.2 mm; ML, ±1.5 mm; DV, −4.0 mm. For the second coordinates: AP, +4.2 mm; ML, ±1.5 mm; DV, −5.0 mm. The third, fourth and fifth pairs of coordinates were AP, +4.2 mm, ML, ±1.5 mm, and DV, −3.0 mm; AP, +3.4 mm, ML, ±1.7 mm and DV, −4.0 mm; and AP, +3.4 mm, ML, ±1.7 mm and DV, −3.0 mm respectively.

For the midline lesion, the needle was positioned at the same AP and DV coordinates as used for the MDmc lesions with the ML set at 0.0 mm from the third ventricle.

Neurotoxic injections to the intended mediodorsal thalamic nuclei in subjects MDP1, MDP2 and MDP3 were produced by 10 (5 per hemisphere) × 1 μL injections of a mixture of ibotenic acid (10 mg/mL; Biosearch Technologies) and N‐methyl‐d‐aspartate receptor (NMDA) (10 mg/mL) dissolved in sterile 0.1 mm PBS. For MDP4, 10 × 1.3 μL injections were made. This mixture of ibotenic acid and NMDA targets NMDA receptors and metabotropic glutamate receptors and has previously produced excellent thalamic lesions in rhesus macaques (Mitchell *et al*., 2007a,b, 2008; Mitchell & Gaffan, 2008; Browning *et al*., 2015; Chakraborty *et al*., 2016). Each injection was made slowly over 4 min and the needle was left in place for 4 min before being moved to the next site.

When the lesion was complete, the dura was repositioned but not sewn, the bone flap was replaced and held with loose sutures, and the galea and skin were closed with sutures in layers. To reduce cerebral oedema, mannitol (20%; a sugar alcohol solution; 1 mg/kg, i.v.) was administered slowly for 30 min when the monkey was still anaesthetised. Then, the monkey was removed from the head holder and anaesthesia discontinued. The monkey was extubated when a swallowing reflex was observed, placed in the recovery position in a cage within a quiet, darkened room and monitored continuously. Normal posture was regained upon waking (waking times varied between 10 and 40 min after the discontinuation of anaesthesia). Recovery went well for all animals, although the neurotoxic injections caused sleepiness in all the four animals for up to 48 h postoperation; all monkeys were kept warm with blankets and appropriate fluid and food intake was maintained during this time. Operated monkeys re‐joined their socially housed environment as soon as practical after surgery, usually within 3–5 days of the operation.

Postoperative medication continued in consultation with veterinary staff, including steroids (dexamethasone, 1 mg/kg, i.m.), the dose was once every 12 h for 4 days, then once every 24 h for 3 days; analgesia (buprenorphine, 0.01 mg/kg, i.m.) for 48 h; and antibiotic treatment (amoxicillin, 8.75 mg/kg, oral) for 5 days. Gastric ulcer protection (omeprazole, 5 mg/kg, oral; and antepsin, 500 mg/kg, oral) commenced 2 days prior to surgery and continued postoperatively for the duration of other prescribed medications, up to 7 days.

#### Histology

After completion of all behavioural testing each monkey was sedated with ketamine (10 mg/kg), deeply anaesthetised with intravenous barbiturate and transcardially perfused with 0.9% saline followed by 10% formalin. The brains were cryoprotected in formalin sucrose and then sectioned coronally on a freezing microtome at 50 μm thickness. A 1‐in‐10 series of sections was collected throughout the cerebrum that was expanded to a 1‐in‐5 series through the medial thalamus. All sections were mounted on gelatin‐coated glass microscope slides and stained with cresyl violet.

### Statistical analysis

For Experiment 1, repeated‐measures *t*‐tests (paired samples) with significance set at *P *<* *0.05 were used to compare mean per cent errors for each of the different surgical lesioned animals in their preoperative vs. postoperative performance tests (Posttest1 and Posttest2). Single case analyses (Crawford & Howell, 1998) were also performed for each monkey against the normative sample (preoperative performance) to assess abnormality of test scores. For Experiment 2, non‐parametric statistics were used to compare the mean difference scores across Test 1 and Test 2 during the food reward devaluation critical test sequences. Single case analyses were also performed for each monkey against the normative sample [unoperated control monkey performance previously published by Mitchell *et al*. (2007b)] to assess abnormality of test scores (Crawford & Howell, 1998).

## Results

### Assessment of the MDpc/MDmc lesions

**Figure 2:**
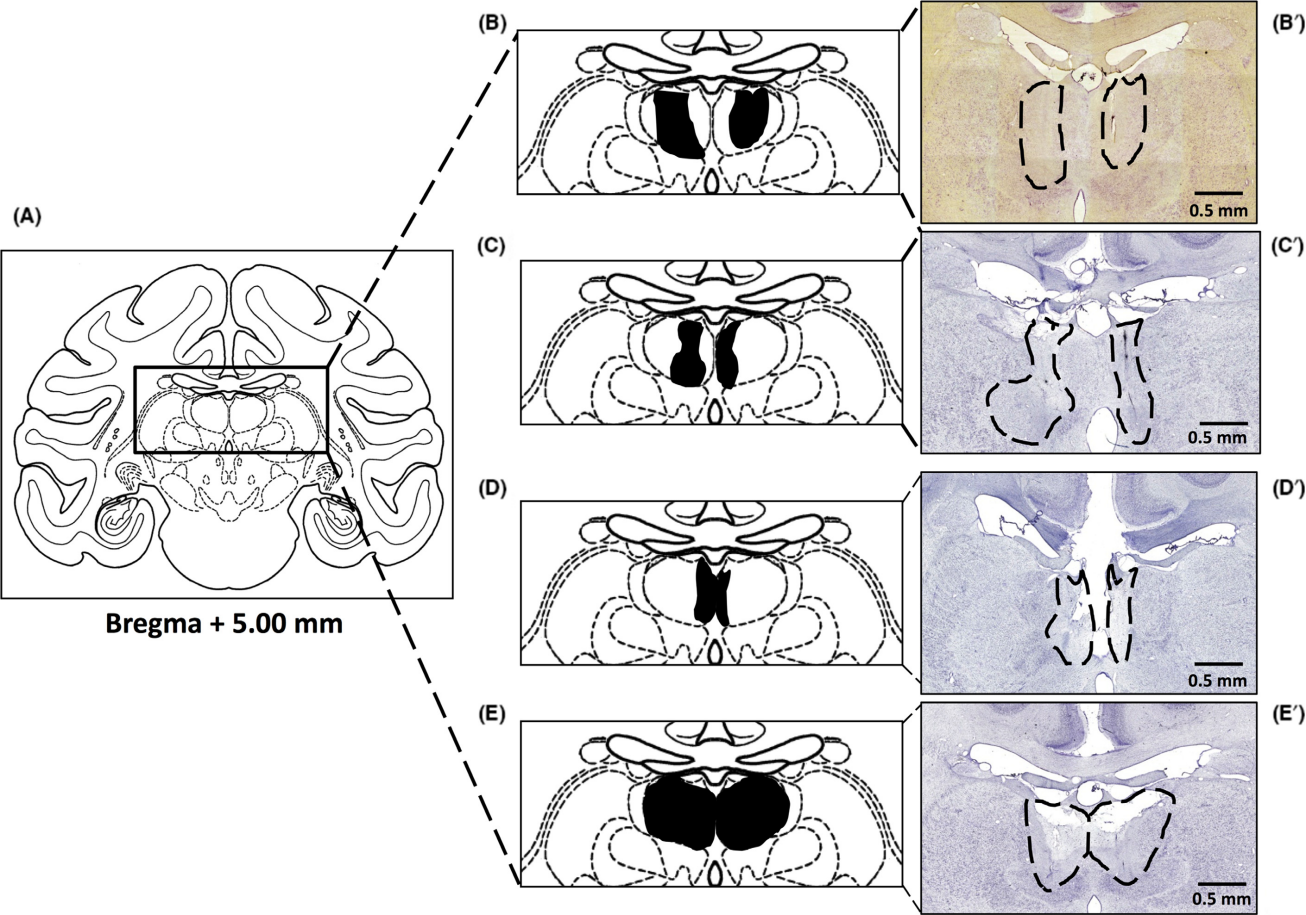
Histology. Left: (A) a schematic coronal section from our rhesus monkey atlas showing the dorsal thalamus. Middle: Enlarged coronal sections of the mediodorsal thalamus showing the extent of the damage to the parvocellular (MDpc) and magnocellular (MDmc) subdivisions in the four rhesus macaque monkeys with neurotoxic lesions. (B) MDP1, with bilateral MDpc damage; (C) MDP2, with combined unilateral damage to the MDpc (left hemisphere) and MDmc in contralateral hemispheres; (D) MDP3, with unilateral MDmc (left hemisphere) and midline damage; (E) MDP4, with bilateral damage to both the MDpc and MDmc. Right: Photomicrographs of the actual neurotoxic lesion to (B′) MDP1; (C′) MDP2; (D′) MDP3 and (E′) MDP4.

Table 1 shows the percentage of lesion damage to MDpc and to the MDmc in each of the four monkeys (MDP1, MDP2, MDP3 and MDP4) participating in the current experiments. In addition, Fig. 2 shows schematic diagrams of the damage to the mediodorsal thalamus for each of the four monkeys as well as photomicrographs of cresyl violet stained coronal sections corresponding as closely as possible to the schematic diagrams for the four monkeys with damage to the MDpc and MDmc. Monkey MDP1 had bilateral MDpc lesions. MDP2 had unilateral MDpc (left hemisphere) and MDmc damage in opposite hemisphere (right). MDP3 had unilateral damage (left hemisphere) to the MDmc and midline. MDP4 had extensive bilateral MDpc and MDmc damage. All animals also had sagittal section of the splenium of the corpus callosum (note that this small sectioning alone does not affect performance on the scene learning task as tested in surgical controls; see Parker & Gaffan, 1997; Mitchell & Gaffan, 2008; Browning *et al*., 2015).

**Table 1:** Per cent of lesion damage to MDpc and MDmc

Monkey	MDpc – left hemisphere	MDpc – right hemisphere	MDmc – left hemisphere	MDmc – right hemisphere
MDP1 – Bi MDpc	100%	98.1%	0	0
MDP2 – Uni MDpc × Uni MDmc	99.6%	0	0	65.3%
MDP3 – Uni MDmc + midline	0	0	92.4%	0
MDP4 – Bi MDpc + Bi MDmc	100%	100%	100%	100%

The extent of the neurotoxic lesion damage for each animal has been quantified using image j software.

### Experiment 1: object‐in‐place discrimination learning after MDpc lesions

**Figure 3:**
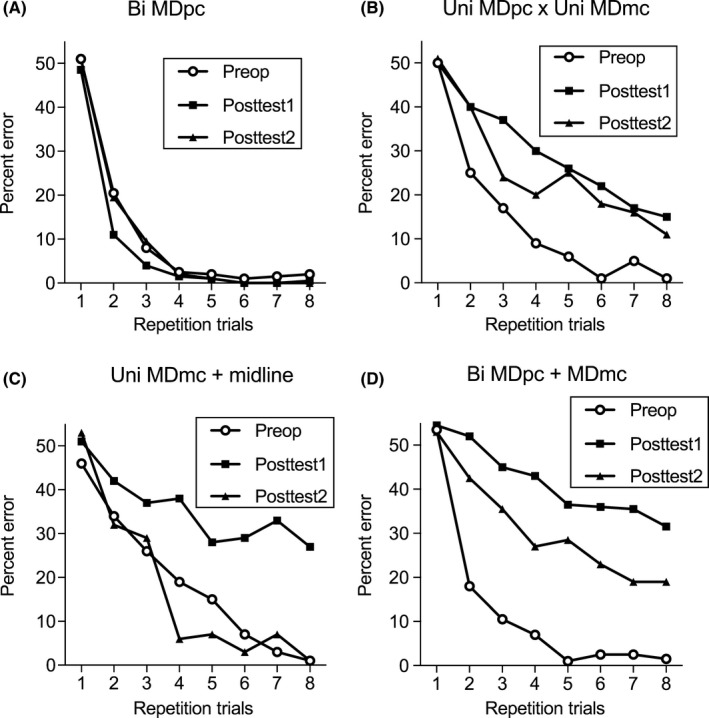
Mean per cent error of performance across learning trials in the object‐in‐place learning discrimination task prior to surgery (open squares) and immediately after postsurgery recovery during postoperative test 1 (Posttest1: black squares) and after completing Experiment 2 during postoperative test 2 (Posttest2: black triangles). (A) Monkey MDP1, after bilateral neurotoxic lesions to the parvocellular nucleus of the mediodorsal thalamus (MDpc); (B) monkey MDP2, after a combined unilateral (left) neurotoxic lesion to the MDpc and contralateral unilateral neurotoxic lesion to the magnocellular subdivision of the MD (MDmc); (C) monkey MDP3, after a combined unilateral (left) neurotoxic lesion to the MDmc and midline of the thalamus; (D) monkey MDP4, after a combined bilateral neurotoxic lesion to both the MDpc and to the MDmc.

**Figure 4:**
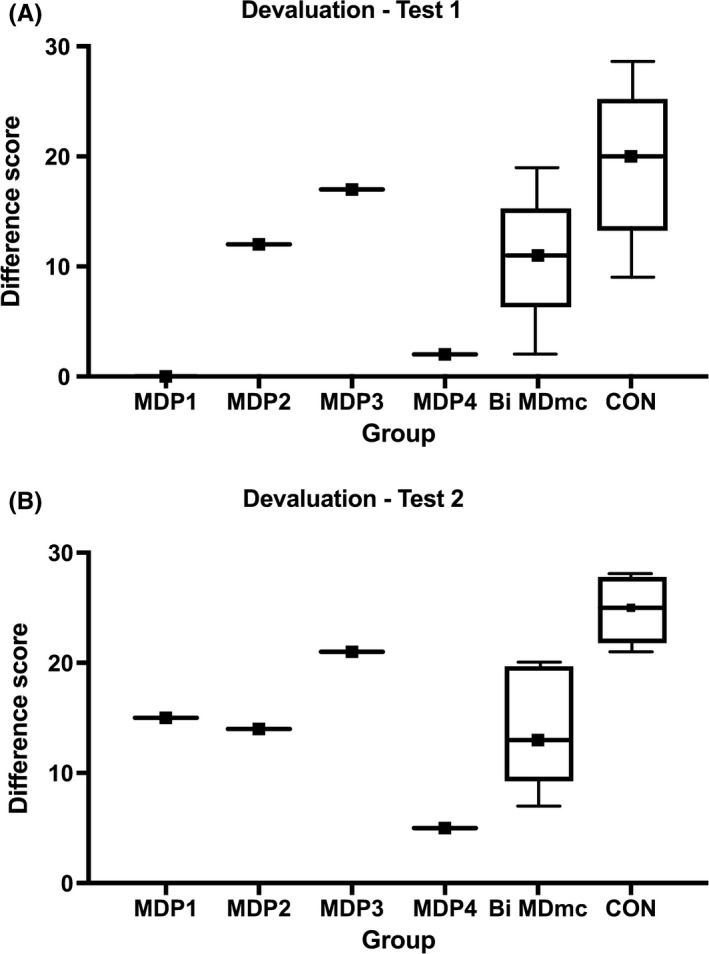
Devaluation. Box and whisker plots (A) Devaluation Test 1 and (B) Devaluation Test 2 for individual monkeys, MDP1, after bilateral neurotoxic lesions to the parvocellular nucleus of the mediodorsal thalamus (MDpc); MDP2, after a combined unilateral (left) neurotoxic lesion to the MDpc and contralateral unilateral neurotoxic lesion to the magnocellular subdivision of the MD (MDmc); MDP3, after a combined unilateral (left) neurotoxic lesion to the MDmc and midline of the thalamus; MDP4, after a combined bilateral neurotoxic lesion to both the MDpc and to the MDmc: Bi MDmc, bilateral neurotoxic lesions to the MDmc, previously published in Mitchell *et al*. (2007a,b); CON, unoperated control monkeys, previously published in Mitchell *et al*. (2007a,b). Box shows the median and upper and lower quartiles, whiskers show the range.

The four monkeys with neurotoxic lesions to the MDpc, or MDpc and MDmc, or the MDmc and midline learned new object‐in‐place scene problems in each session. Monkeys, MDP1 and MDP4, learnt 20 new object‐in‐place scene problems per session, while monkeys, MDP2 and MDP3, learnt 10 new object‐in‐place scene problems per session. The dependent measure was the mean per cent errors made during rapid within‐session learning of new object‐in‐place scene discriminations repeated 8 times per session averaged across 10 consecutively ran testing sessions during the preoperative (Pre) and postoperative (Posttest1; Posttest2) performance tests. The first postoperative test (Posttest1) was performed immediately after postoperative recovery (up to 14–19 days postsurgery). The second postoperative test (Posttest2) was performed after completing Experiment 2 (up to 90–150 days postsurgery). The data from each of the four monkeys performing in these tests are shown in Figs 3 and 4 and Table 2.

**Table 2:** Mean per cent errors in trial blocks 2–8 in object‐in‐place scene discrimination learning

Monkey	Pre – % error	Posttest1 – % error	Single case analysis*	Deficit = pre – posttest1	Posttest2 – % error	Single case analysis*	Deficit = pre – posttest2
MDP1 – Bi MDpc	5.36	2.57	*P* = 0.143	+2.79	4.57	*P *= 0.220	+0.79
MDP2 – Uni MDpc × Uni MDmc	9.14	26.71	*P* = 0.018	−17.57	22	*P* = 0.038	−12.86
MDP3 – Uni MDmc + midline	15.00	33.86	*P* = 0.007	−18.86	12.14	*P* = 0.278	+2.86
MDP4 – Bi MDpc + Bi MDmc	6.14	39.93	*P* = 0.004	−33.79	27.78	*P* = 0.015	−21.64
Uni MDmc (Browning *et al*., 2015)	7.41 (4.59)	23.90 (5.33)	×	−16.50 (0.82)	×	×	×
Bi MDmc (Mitchell *et al*., 2007a,b)	5.78 (2.43)	32.50 (13.86)	×	−26.72 (16.28)	×	×	×

Object‐in‐place scene learning mean (and standard deviation) per cent error rates are shown for individual rhesus macaque monkeys (MDP1, MDP2, MDP3 and MDP4) before and after neurosurgery to produce differing neurotoxic lesions to the parvocellular subdivision (MDpc) or the magnocellular subdivision (MDmc) of the mediodorsal thalamic nucleus (MD). Additional unilateral MDmc and bilateral MDmc neurotoxic lesion groups are presented for comparison; *Single case analyses presented using the normative sample is preoperative test (*M* = 8.91, SD = 4.37, *N* = 4).

For monkey MDP1, selective bilateral MDpc lesions did not impair postoperative learning of object‐in‐place scene discriminations. A repeated‐measures *t*‐test of the errors made during the preoperative (Pre: *M* = 5.36, SD = 2.50) vs. postoperative (Posttest1: *M* = 2.57, SD = 1.22) performance tests for this one animal demonstrated significantly improved performance after the neurosurgery [*t*_(9)_ = 3.48, *P *=* *0.007; Fig. 3]. During Posttest2, the average per cent errors made across the 10 test sessions (Posttest2: *M* = 4.57, SD = 3.39) also did not differ to the preoperative performance test [*t*_(9)_ = 0.53, *P *=* *0.609; Fig. 3]. This causal evidence, although only established in one animal with near‐complete MDpc bilateral lesions (see Table 1), indicates that bilateral MDpc neurotoxic damage by itself is not enough to impair rapid within‐session new learning of object‐in‐place scene discriminations.

In contrast, the monkey, MDP4, with the large bilateral damage to both the MDpc and the MDmc was impaired during postoperative learning of the object‐in‐place scene discriminations compared to his own preoperative performance. A repeated‐measures *t*‐test of the average per cent errors made by MDP4 during the preoperative (Pre) vs. postoperative (Posttest1) performance tests confirmed that the additional errors made after surgery (*M* = 39.93, SD = 7.07) were significantly different from his preoperative performance (*M* = 6.14, SD = 3.12) [*t*_(9)_ = 19.75, *P *=* *0.001; Fig. 3]. During the Posttest2 performance test, the average per cent errors made (Posttest2: *M* = 27.79, SD = 8.88) were also significantly different to the average per cent errors made during the preoperative performance test [*t*_(9)_ = 7.35, *P *=* *0.001; Fig. 3]. Furthermore, a comparison of the average per cent errors made during Posttest2 vs. Posttest1 indicated a significant reduction in the number of errors made during Posttest2 [*t*_(9)_ = 3.29, *P *=* *0.009; Fig. 3]. Nevertheless, the extent of the severity of this new learning impairment in MDP4 was similar to that seen in monkeys from an earlier study using exactly the same task with bilateral MDmc lesions caused by neurotoxic injections (Mitchell *et al*., 2007a).

For monkey MDP2, the selective unilateral lesions to the MDpc and the MDmc in opposite hemispheres of the thalamus caused the monkey to make more errors during postoperative learning of object‐in‐place scene discriminations. A repeated‐measures *t*‐test of the average per cent errors made during the preoperative (Pre: *M* = 9.14, SD = 5.39) vs. postoperative (Posttest1: *M* = 26.71, SD = 8.83) performance tests for this one animal demonstrated a significant impairment after the neurosurgery [*t*_(9)_ = 7.07, *P *=* *0.001; Fig. 3]. During the Posttest2 performance test, the number of errors made (Posttest2: *M* = 22.00, SD = 10.63) was also significantly different from the monkeys’ own preoperative performance test [*t*_(9)_ = 3.50, *P *=* *0.007; Fig. 3]. However, in this monkey, a comparison of the average per cent errors made during Posttest2 vs. Posttest1 indicated no significant reduction in the errors made during Posttest2 [*t*_(9)_ = 1.65, *P *=* *0.133; Fig. 3]. The extent of the severity of impairment in MDP2 was similar to that seen in an earlier study using exactly the same task with unilateral MDmc lesions caused by neurotoxic injections (Browning *et al*., 2015), suggesting that the unilateral MDmc damage was causing the rapid within‐session new learning impairment.

Lastly for monkey MDP3, the selective unilateral lesion to MDmc combined with a midline lesion also impaired postoperative learning of object‐in‐place scene discriminations. A repeated‐measures *t*‐test of the errors made by MDP3 in the preoperative (Pre) vs. postoperative (Posttest1) performance tests confirmed that the additional errors made during learning after surgery (*M* = 33.86, SD = 12.33) were significantly different from preoperative performance (*M* = 15.00, SD = 7.20) [*t*_(9)_ = 3.81, *P *=* *0.004; Fig. 3], reconfirming that unilateral damage to the MDmc is sufficient to impair new object‐in‐place within‐session learning (Browning *et al*., 2015). Interestingly though, in the second postoperative test (Posttest2), performed many months after the initial neurosurgery and after the completion of Experiment 2, the performance of monkey MDP3 improved substantially, as he made less errors than in his preoperative test. During the Posttest2 performance test, the average per cent errors made (Posttest2: *M* = 12.14, SD = 8.68) did not differ from the average per cent errors made during the preoperative performance test [*t*_(9)_ = 1.12, *P *=* *0.291: Fig. 3], suggesting that recovery of function had occurred after the selective unilateral MDmc/midline lesion with repeated cognitive training in other tasks and subsequent testing.

### Experiment 2: object–reward association learning to criterion and reinforcer devaluation

#### Object–reward association learning

The monkeys with differing damage to the MDpc and MDmc typically learned the rewarded object discrimination problems at a similar rate, except for monkey MDP3 [see Table 3 for details of individual sessions to criterion (not including the criterion run) and mean errors to criterion (not including the criterion run) for each monkey]. Monkey MDP3 was slower to learn the associations compared with the other three monkeys; he attained his first 90% correct level by the 37th session of training, however, he continued to display inconsistent learning of the object–reward association pairings, until he reached 66 training sessions and had scored over 1000 errors (his task learning endpoint had been set at 1000 errors). For the other three monkeys (MDP1, MDP2, and MDP4) their training sessions to criterion and errors were very similar to unoperated control monkeys from a previously published study (Mitchell *et al*., 2007b) that learned the exact same stimuli in this object–reward association task [*n *=* *4, errors, *M* = 248.75 (SD ±136.87) and sessions, *M* = 15.75 (SD ±9.07)].

**Table 3:** Object–reward association learning and devaluation performance for the two tests

Monkey	Object‐reward association		Devaluation task – Test 1					Devaluation task – Test 2				
	Errors to criterion	Sessions to criterion	Baseline M : P	Satiate M M : P	Satiate P M : P	Diff score	Single case analysis†	Baseline M : P	Satiate M M : P	Satiate P M : P	Diff score	Single case analysis‡
MDP1 – Bi MDpc	141	11	29.5 : 0.5	30 : 0	30 : 0	0	*P *= 0.060	29.5 : 0.5	15 : 15	30 : 0	15	*P* = 0.039
MDP2 – Uni MDpc × Uni MDmc	242	11	28.5 : 1.5	18 : 12	30 : 0	12	*P *= 0.238	26.5 : 3.5	13 : 17	27 : 3	14	*P* = 0.031
MDP3 – Uni MDmc + midline	1004*	66*	17 : 13	8 : 22	25 : 5	17	*P *= 0.408	14.5 : 15.5	22 : 8	1 : 29	21	*P* = 0.192
MDP4 – Bi MDpc + Bi MDmc	203	10	18 : 12	11 : 19	13 : 17	2	*P *= 0.074	16.5 : 13.5	16 : 14	21 : 9	5	*P* = 0.006

The number of M&M (M) and peanut (P) objects chosen by each monkey during the baseline sessions (mean of two baseline sessions for each test) and for sessions preceded by devaluation of either peanuts or M&Ms. *MDP3 was slower to learn the associations compared with the other three monkeys (refer to results for details). ^†^Individual difference scores for Test 1 compared to the normative sample of unoperated controls from Mitchell *et al*. (2007a,b): *M* = 19.25, SD = 7.97 and *N* = 4; ^‡^Individual difference scores for Test 2 compared to the normative sample of unoperated controls from Mitchell *et al*. (2007a,b): *M* = 24.75, SD = 3.30 and *N* = 4.

#### Devaluation

The performance data on the two devaluation tests from the monkeys with differing damage to the MDpc and MDmc are also presented in Table 3. The difference scores on each test were the main dependent measure of reward devaluation performance, with smaller positive difference scores representing attenuated decision‐making after food value (satiety) devaluation. The difference scores were calculated as the difference in the number of choices of objects paired with a particular reward on the baseline sessions and in sessions when that food reward was devalued. These scores were added together for each devalued food during the critical test sequences for each test. The overall mean difference score was the average of the difference scores on Test 1 and Test 2. The difference scores of all of the monkeys with different amounts of MD damage were higher for the second devaluation test (*M* = 13.50, SD = 7.05) than the first (*M* = 7.75, *SD* = 8.09), which is congruent with the previous investigations using this task in both our own and in other laboratories (Málková *et al*., 1997; Baxter *et al*., 2000; Izquierdo *et al*., 2004; Mitchell *et al*., 2007b; Izquierdo & Murray, 2010; Browning *et al*., 2015). A non‐parametric Wilcoxon Signed Rank repeated‐measures *t*‐test using the entire sample of monkeys (MDP1, MDP2, MPD3 and MDP4) confirmed that the mean difference scores for the two devaluation tests were not significant (*Z *=* *1.84, *P *=* *0.066).

Given that our current devaluation performance data cannot be combined into different groups, we have instead presented each monkey (MDP1, MDP2, MDP3 and MDP4) individually and compared their performance to previously published unoperated control monkeys (CTLs) (Mitchell *et al*., 2007b) using single case study analysis (Crawford & Howell, 1998). During the first devaluation test, while monkeys with differing damage to the MDpc and MDmc all eventually learned the discrimination problems that formed the critical trials for the devaluation test, MDP1, MDP2 and MDP4 showed attenuated devaluation scores compared to the previously published unoperated controls, as indicated in Table 3 and in Fig 4A and B, while MDP3 with unilateral MDmc and midline damage had a difference score that was similar to the unoperated controls. However, the single case analyses conducted for each individual monkey compared to the unoperated control monkey normative sample were all non‐significant (*P*'s > 0.05; see Table 3 for individual *p* values). This lack of significant differences between each individual lesioned animal and the normative control group is likely the result of the more variable difference scores produced in the first devaluation test by the unoperated control group (see Figure 4A and B).

In contrast, during the second devaluation test, monkeys with differing damage to the MDpc and MDmc (MDP1, MDP2 and MDP4) showed attenuated devaluation scores compared to the unoperated controls (*P*'s = 0.039 or less), as indicated in Table 3 and in Fig. 4B, while MDP3 continued to have a difference score that was not different (*P* = 0.192) from the unoperated controls.

Given that some of the monkeys (MDP1, MDP2 and two of the controls) displayed a clear preference for M&M rewards in both the baseline sessions and in the devaluation tests, we conducted a further analysis that was restricted to sessions linked to the M&M devaluation tests only. Thus in this further analysis, the difference scores were calculated as the difference in the number of choices of objects paired with M&M reward on the baseline sessions and in sessions when that M&M food reward was devalued. The difference scores were higher for the second devaluation test (*M* = 10.50, SD = 6.68) than the first (*M* = 0.37, SD = 0.32), which is congruent with the previous investigations using this task. These scores were added together. The overall mean difference score was the average of the difference scores on Test 1 and Test 2. A non‐parametric Wilcoxon Signed Rank repeated‐measures *t* test using the entire sample of monkeys (MDP1, MDP2, MDP3 and MDP4) confirmed that the mean difference scores for the two devaluation tests were not significant (*Z* = 1.82, *P* = 0.125).

By comparison, the overall mean difference score from unoperated control monkeys showed that they did devalue the food reward that had been satiated just prior to the critical test sessions (*M* = 12.75, ±6.66). The overall mean difference score from the two tests of devaluation to M&M for the two groups (Control and MDpc) was compared using non‐parametric Mann–Whitney *U*‐independent samples *t*‐test, and the results showed that there was no difference between the two groups (*P* > 0.05).

**Figure 5:**
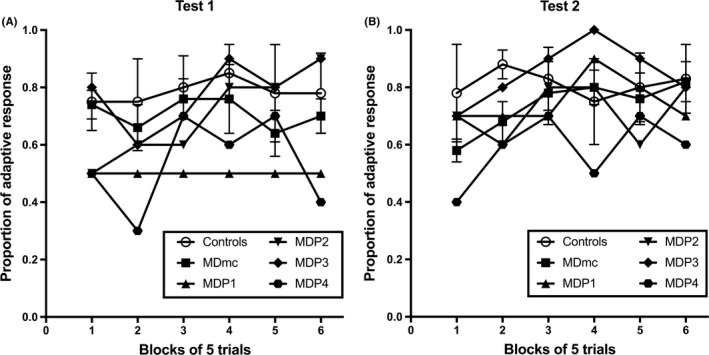
Devaluation. Adaptive responding. Mean probabilities of adaptive responses made during (A) Devaluation Test 1 and (B) Devaluation Test 2 for individual monkeys after bilateral neurotoxic lesions to the parvocellular nucleus of the mediodorsal thalamus (MDpc); MDP2, after a combined unilateral (left) neurotoxic lesion to the MDpc and contralateral unilateral neurotoxic lesion to the magnocellular subdivision of the MD (MDmc); MDP3, after a combined unilateral (left) neurotoxic lesion to the MDmc and midline of the thalamus; MDP4, after a combined bilateral neurotoxic lesion to both the MDpc and to the MDmc: Bi MDmc, bilateral neurotoxic lesions to the MDmc, previously published in Mitchell *et al*. (2007a,b); CON, unoperated control monkeys, previously published in Mitchell *et al*. (2007a,b).

To further explore possible individual lesion differences in adaptive decision‐making, the proportion of adaptive responses made on the 30‐trial devaluation test sessions for each MDpc monkey (MDP1, MDP2, MDP3 and MDP4) and the two groups of monkeys with bilateral MDmc lesions or unoperated controls previously published in Mitchell *et al*. (2007b) were computed. Figure 5A and B show the proportion of adaptive responses after selective satiation for Test 1 and Test 2 respectively. This measure, unlike the difference score, is independent of the baseline data. To compute the proportion of adaptive responses, each trial of the 30‐trial devaluation test that followed the selective satiation was scored with either 1 or 0. A score of 1 was recorded when the chosen object was associated with the non‐satiated food, whereas a score of 0 was recorded when the chosen object was associated with the devalued food. Thus, the monkeys with more adaptive responses had scores closer to 1. Data from the two sessions (1 after devaluation of each food type) within each test were averaged and then divided into six blocks of five trials each.

A 6 (trial block: 1, 2, 3, 4, 5 and 6) × 2 (group: MDmc and CON) mixed‐model repeated‐measures ANOVA assessed the adaptive responses in each test and revealed no main effect of Trial block (*F* < 1.0), group (*F* < 1.0) and no significant interaction of Trial block × Group, (*F* < 1.0) for Test 1 (Fig. 5A). For Test 2, the anova also showed no main effect of Trial block, *F*_5,35_ = 1.07, *P *=* *0.394, group, (*F* < 1.0) and no significant interaction of Trial block × Group, *F*_5,35_ = 1.10, *P* = 0.379. Interestingly though, for the within‐subject contrasts in Test 2, there was a linear trend for a Trial Block x Group interaction, *F*_1,7_ = 4.07, *P* = 0.084 (Fig. 5B) indicating that the monkeys with bilateral MDmc lesions required further trials, as indicated in Fig. 5B, up to 15 trials to adapt their decisions as measured by their behavioural responses during the critical test. No statistical analyses were performed on the single lesioned animals, although their individual averaged adaptive response data for Test 1 and for Test 2 are plotted for comparison.

As detailed in Fig. 5A and B, the proportions of adaptive responses shown from the monkeys with differing lesions to the MDpc and MDmc indicate that typically they were performing at a level indistinguishable from each other on the food reward devaluation task. However, as is also evident in the figures, across both Test 1 and Test 2, the monkey (MDP4) with the large bilateral lesions to both the MDmc and MDpc consistently made the least adaptive responses across the 30 trials in each of the 30‐trial devaluation adaptive decision‐making tests.

Finally, the amounts of time spent completing the devaluation procedure were similar across the different monkeys (MDP1, 12 mins; MDP2, 15½ mins; MDP3, 12 mins; MPD4, 13¾ mins) and these times were similar to previously published data from the unoperated controls and monkeys with bilateral MDmc lesions (Mitchell *et al*., 2007b).

## Discussion

This study presents the first research using rhesus macaques to assess the specific contributions of neurotoxic lesions to the MDpc and MDmc in different learning and decision‐making tasks. During rapid within‐session learning in the object‐in‐place scene discrimination task, bilateral damage to the MDpc had little impact, leaving new learning intact. Instead, damage to the MDmc was critical to producing new learning impairments in this task, whether that was unilateral [MDP2 and MDP3; reconfirming the surprising result of unilateral MDmc deficits observed by Browning *et al*. (2015)] or bilateral (MDP4). It must be noted though that these findings are based on single case lesion monkeys so should be regarded as preliminary observations.

In contrast, during the object–reward association task in Experiment 2, that was learnt to a 90% correct performance across five consecutive sessions criterion, with one trial of each object–reward pair presented per session, performance was not disrupted by the neurotoxic MD lesions. One monkey, MDP3, was slower than the other monkeys with MDmc or MDpc lesions, to attain the 90% performance across five consecutive sessions criterion. However, his ability to learn was likely hampered by his lack of consistent, stable performance during daily training sessions rather than it being due to the unilateral MDmc/midline lesion itself.

For the food (satiety) devaluation task in Experiment 2, monkeys with MDpc lesions, like monkeys with MDmc lesions, showed attenuated scores in the devaluation paradigm. Specifically MDP1, with bilateral MDpc lesions, and MDP2, with combined unilateral MDpc and MDmc damage across hemispheres, showed attenuated devaluation effects after satiety. While monkey MDP4 with the combined bilateral damage to both the MDmc and MDpc showed the most attenuated food reward devaluation effect and the least adaptive responding across both 30‐trial devaluation tests (Fig. 5A and B). Electrophysiological recordings in monkeys have indicated that neurons throughout the OFC are most closely implicated in representing the reward value of stimuli or outcomes (Tremblay & Schultz, 1999; Roesch & Olson, 2007). Furthermore, selective bilateral OFC lesions cause attenuated food reward devaluation in monkeys using this same touch screen–based adaptive decision‐making task (Baxter *et al*., 2007). The MDmc, the MD subdivision most heavily interconnected with the medial OFC and recipient of basolateral amygdala inputs (Xiao *et al*., 2009; Timbie & Barbas, 2015) has already been shown to have a functional role in this reward devaluation task (Mitchell *et al*., 2007b; Izquierdo & Murray, 2010; Browning *et al*., 2015). In addition, MDpc is reciprocally connected with lateral areas of the OFC (McFarland & Haber, 2002). Now, this current evidence indicates, from these first preliminary observations, a functional role of the MDpc in food reward (satiety) devaluation. Thus, the most parsimonious explanation for the similarity of attenuated food devaluation effects following either or both MDmc or MDpc damage would be that the reciprocal interactions between the OFC and both the MDmc and MDpc are sufficient to disrupt optimal performance in this adaptive decision‐making task.

However, for the object‐in‐place learning task, the dissociation deficits are perhaps harder to reconcile. Nevertheless, these preliminary results, obtained in single case lesioned monkeys, do suggest that it is more likely to be the MDmc damage that is markedly disrupting the monkeys’ ability to learn new object‐in‐place scene discriminations. In addition, the lack of new learning impairments for the monkey (MDP1) with bilateral neurotoxic MDpc damage suggests again, as previously reported (Browning *et al*., 2015) that the surgical approach, the surgical procedure and the postsurgical recovery are not likely to be the cause of the learning deficits for the other monkeys with unilateral or bilateral MDmc damage. Furthermore, and critically, for the reward devaluation task, either bilateral damage to the MDmc or the MDpc, or a combination of damage to both MD subdivisions is sufficient to disrupt adaptive decision‐making performance after food reward satiety, indicating dissociable cognitive performance across these two tasks that is likely governed by their unique interconnectivity with other cortical and subcortical brain structures.

Thus, this preliminary evidence of some specialisation of cognitive functioning to different subdivisions of the MD, with MDpc and MDmc demonstrating dissociable performance in these two cognitive tasks, has similarities to previously documented evidence detailing dissociable cognitive abilities across many different tasks among the many different areas of the frontal cortex (Passingham & Wise, 2012). For example, bilateral DLPFC ablations to area 9 and area 46 including both dorsal and ventral banks (frontal areas interconnected to the MDpc) leaves intact both new learning of object‐in‐place scene discrimination learning and reward devaluation after satiety performance (Baxter *et al*., 2008b). On the other hand, selective bilateral damage to other cortical frontal areas, for example, to either the ventrolateral prefrontal cortex or to the OFC impairs performance in the object‐in‐place scene learning task, whether the lesions are caused by ablations (Baxter *et al*., 2007, 2008a) or neurotoxic injections (Wilson *et al*., 2007). However, for reward devaluation, as indicated above, bilateral OFC lesions cause attenuated food devaluation choices in this touch screen paradigm (Baxter *et al*., 2007) and in experimenter‐controlled hand‐fed food reward devaluation paradigms (Málková *et al*., 1997; Baxter *et al*., 2000; Izquierdo & Murray, 2004), while bilateral ventrolateral prefrontal (area 45) cortex lesions leave performance intact (Baxter *et al*., 2009). Thus, to explain the dissociable deficits in learning after MDmc or MDpc damage compared with similar deficits in adaptive decision‐making, we must review evidence from damage in wider neural networks to potentially understand the underlying mechanism involved.

For the object‐in‐place scene discrimination learning task, interactions between the inferotemporal cortex and the frontal cortex have also been shown to be critical to support new learning performance, whether the damage involves crossed hemisphere unilateral disconnections of the inferotemporal cortex and prefrontal cortex, or uncinate fascicle white matter pathway ablations (Browning & Gaffan, 2008a,b; Gaffan & Wilson, 2008). Human neuroimaging studies are now also pointing to the importance of the uncinate fascicle, the white matter pathway connecting the inferotemporal cortex to the inferior convexity and orbital surface of the prefrontal cortex (Ungerleider *et al*., 1989) for learning visual associations (Thomas *et al*., 2015). Furthermore, in patients with mild cognitive impairment, a precursor to Alzheimer's disease, and in amyotrophic lateral sclerosis patients with memory deficits, changes in the integrity of the uncinate fascicle have been correlated with the extent of the memory decline (Christidi *et al*., 2017; Liu *et al*., 2017; Wang *et al*., 2017). Finally, recent neuroimaging evidence from macaque monkeys has also demonstrated areas activated within a temporal–prefrontal cortex network during learning and long‐term memories for object–reward associations (Ghazizadeh *et al*., 2018). Interestingly though, neuroanatomical tracing studies in primates have indicated that the MDpc does not receive inputs from the temporal lobes, including the inferotemporal cortex. In contrast, primate MDmc receives monosynaptic inputs from the rhinal cortex, which is part of the inferotemporal cortex, and the amygdala (Russchen *et al*., 1987; Saunders *et al*., 2005; Timbie & Barbas, 2015). Thus, while these new results from single case monkeys must be considered as preliminary observations of the dissociable effects in learning rapid visual associations in the object‐in‐place scene discrimination task, the neuroanatomical network connectivity differences between the MDpc and MDmc are also supportive of this dissociable learning and memory performance. That is, the frontal‐temporal neural network connecting ventrolateral and orbital prefrontal cortex with the inferotemporal cortex, and involving a transthalamic route of communication via the MDmc, but not the MDpc, may be particularly important for rapid within‐session new learning of object‐in‐place visual associations in primates.

An interesting observation in this study was the recovery of function across time in our monkey MDP3. MDP3 had unilateral MDmc combined with midline damage and showed impairments in object‐in‐place scene discrimination learning during the first test (Posttest1) that had returned to his preoperative performance levels by the second postoperative test (Posttest2) suggesting that recovery of functioning can occur after this more selective unilateral damage to the midline and adjacent MDmc area. In a previous study from our laboratory (Browning *et al*., 2015), unilateral MDmc lesions also caused new learning deficits in the object‐in‐place scene discrimination learning task, as reported above. In contrast though, in the same study, unilateral combined ablations of the ventrolateral prefrontal cortex and OFC lesions left new learning intact. This observation suggests two things: firstly, that the other intact contralateral hemisphere is sufficient to compensate for the unilateral cortical ablations, and secondly, that the transthalamic route of communication via the MDmc is critical for rapid within‐session learning. We know that the MDmc influences several cortical networks as indicated by the widespread distribution of cortical terminal sites after juxtacellular labelling of single neurons of the medial MD in the rodent brain (Kuramoto *et al*., 2017). Moreover, the monkeys with unilateral damage to the MDmc in the Browning *et al*. (2015) study went on to receive a second lesion involving a selective cortical ablation to the ventrolateral prefrontal cortex and OFC. For the monkeys that received contralateral (CONTRA) lesion damage, they continued to show deficits in learning. However, for the monkeys that received ipsilateral (IPSI) cortical ablations, their performance improved although never fully recovered. Taking all of this evidence together, with the improved performance in MDP3, these preliminary observations are supportive of the notion that the brain can gain some recovery of function over time after unilateral subcortical damage, even though this damage is initially detrimental to learning and memory performance.

In addition, MDP3 did not produce attenuated devaluation scores. Previous evidence has established that contralateral hemisphere interactions between the MDmc and frontal cortex (Browning *et al*., 2015), or among the MDmc, frontal cortex and amygdala (Izquierdo & Murray, 2010), are critical to produce attenuated food devaluation effects, indicating the need to disrupt widespread neural networks across hemispheres. So while these studies demonstrate the differing contributions of the different subdivisions of the MD (MDpc, MDmc) to learning, memory and adaptive decision‐making processes, more critically, they establish that the contributions of the MDmc and MDpc are distinct to those of their immediately reciprocal interconnected cortical areas, and instead highlight the importance of interactions across wider frontal cortical and frontal‐temporal cortical networks that involve differing subdivisions of the MD for certain kinds of learning, memory and adaptive decision‐making. Thus, this behavioural and cognitive evidence combined with the neuroanatomical evidence provides support for the proposals of Guillery and Sherman and colleagues (Sherman & Guillery, 2006; Sherman 2016; Guillery, 2017) that the MDpc and the MDmc are higher‐order thalamic relays based on their input signals received from layer 5 cortical–thalamic projections (Schwartz *et al*., 1991; Timbie & Barbas, 2015), that are then transmitted (or not) via their respective transthalamic routes to help influence the cortical functioning of widely distributed areas and layers of the frontal cortex (Kuramoto *et al*., 2017).

Electrophysiological recordings in monkeys targeting the MDpc have thus far used a delayed oculomotor response (working memory) task to establish the nature of task‐related neuronal signalling. In this task, MDpc neurons show cue‐, delay‐ and response‐period activity, similar to the discharge patterns observed in DLPFC, although most MDpc neurons exhibited a sustained excitatory response during the delay period (Sommer & Wurtz, 2006; Tanibuchi & Goldman‐Rakic, 2005; Watanabe & Funahashi, 2012). While single neurons in the DLPFC have been shown to encode task rules (Wallis *et al*. 2001), some evidence observed during the delay period of the delayed oculomotor task indicates that the retrospective sensory information maintained in the DLPFC may be supplemented by reciprocal inputs to and from the MDpc. Interestingly, it has been shown that these projections help generate prospective motor information and prospective encoding (Funahashi *et al*., 2004; Watanabe & Funahashi, 2012). Furthermore, Watanabe and Funahashi (2012) have proposed that the MDpc is a key area that provides DLPFC with information regarding impending behaviour as response‐period active neurons were more frequent in MDpc than in DLPFC reflecting a bias towards processing motor aspects of the task by the MDpc, which was confirmed further using their population vector analyses. In our experiments, none of the cognitive tasks required only working memory *per se* to perform successfully. However, the food devaluation task requires the monkeys to adjust their choice behaviour in response to changes in the value of one of the rewards (devaluation). This reward devaluation occurs just prior to the critical trial sessions in the food devaluation paradigm. Thus, this value change when signalled in the OFC, may be transferred across cortical routes, and transthalamic routes into the MDmc and MDpc from the OFC, via monosynaptic inputs (McFarland & Haber, 2002) and then rapidly transmitted onto interconnected cortical areas, one of which being the DLPFC via the MDpc.

Cortico‐thalamic projection neurons [from layers 6 (mainly) and 5] to the dorsal thalamus are tenfold more abundant than the thalamocortical inputs to the cortex in adulthood (Jones, 2007). It is suggested that the layer 5 driver projection neurons send via branching axons, efference copies of motor messages to their thalamic recipient terminals that are on the way to motor output centres. These messages are then transmitted (or not – depending on the nature of the neuromodulation from other inputs onto these thalamic terminals) to many other distributed cortical brain regions to update them quickly about upcoming motor responses, via the recipient higher‐order thalamic relay (e.g. MDpc and MDmc). The layer 6 cortico‐thalamic neurons are known to modulate both the firing and excitability of the thalamus, suggesting that the thalamus is not merely a relay of sensory information to the cortex, but is also an important gate through which specific parts of the cortex can communicate rapidly with many other distributed cortical brain regions, via this transthalamic route of information transfer (Guillery, 2017; Sherman & Guillery, 2006, 2013; Sherman, 2016). It still remains to be resolved how and why this duplication of information transfer may exist (i.e. cortico‐cortical and cortico‐thalamo‐cortical transmission) in the mammalian brain and its functional roles in higher‐order cognitive functions. However, it is clear from the behavioural and cognitive evidence produced in animal models so far that the different subdivisions of the MD (MDpc and MDmc) have key roles in supporting this information transfer across differing cortical networks for specific cognitive tasks to rapidly optimise our behavioural choices in order to maximise our rewards.

## Conflict of interest

None of the authors has a conflict of interest to declare.

## Author contributions

ASM designed the study and performed the neurosurgeries; SC, SM and ASM supported the postoperative recovery of the monkeys; SC and SM trained and tested the monkeys; SC, ZO and ASM analysed the data; SC, ZO and ASM wrote the article.

**Abbreviations**
AP: Anteroposterior
CONTRA: contralateral
DLPFC: dorsolateral prefrontal cortex
DV: dorsoventral
Fig: figure
GABA: gamma‐aminobutyric‐acid
ITI: inter‐trial interval
i.m.: intramuscular
i.v.: intravenously
IPSI: ipsilateral
MD: mediodorsal thalamus
MDmc: magnocellular nucleus of the mediodorsal thalamus
MDpc: parvocellular nucleus of the mediodorsal thalamus
MDPn: male monkey number (n) receiving MDpc lesions
ML: mediolateral
NMDA: N‐methyl‐d‐aspartate
OFC: orbitofrontal cortex
Posttest: postoperative test

## Data Availability

The data supporting this manuscript are presented in the manuscript tables.
