# Supplementary material for: Macaque parvocellular mediodorsal thalamus: dissociable contributions to learning and adaptive decision‐making
Source: Eur J Neurosci. 2018 Aug 16;49(8):1041–54. doi: 10.1111/ejn.14078 (PMC6519510; doi:10.1111/ejn.14078)
Supplement: Supplementary file 1 [file EJN-49-1041-s001.docx]

Macaque parvocellular mediodorsal thalamus: dissociable contributions to learning and adaptive decision-making

Mitchell, Anna; Chakraborty, Subhojit; Ouhaz, Zakaria; Mason, Stuart

_____________________________________________________________________________________

| Review timeline: | Submission date: 19.12.17  Editorial Decision: Major Revision  Revision Received:05.05.18  Editorial Decision: Minor Revision  Revision Received: 03.07.18  Accepted: 04.07.18 |  |
| --- | --- | --- |
|  |  |  |

Editor: Paul Bolam

Reviewer 1: Yuri Saalmann

Reviewer 2: Helen Barbas

_____________________________________________________________________________________

1st Editorial Decision	06.02.2018

_____________________________________________________________________________________

Macaque parvocellular mediodorsal thalamus: dissociable contributions to learning and adaptive decision-making

Dear Anna,

Your manuscript has been carefully and thoughtfully reviewed by two external reviewers and the editorial team Both reviewers recognise the importance and significance of your study but both have raise a series of issues that will need to be addressed in a revised version of the manuscript.

Reviewer 1 raises some important points, the most important of which is the issue of the single animals. We, and the reviewer recognise the difficulty and time need to study additional animals which would be way beyond the timescale of this Special Issue. As the reviewer suggests, perhaps you could tone down the conclusions and discuss caveats associated with single animals. Rather than re-drafting as a review as he/she suggests, perhaps put the findings more in a framework of 'preliminary observations'. This reviewer also raise the points about 'ceiling' effects and several other points that will clearly improve your paper. The issues raised by reviewer 2 can mostly be addressed by clarification of the text and inclusion of additional references. He/she points out several errors in Figure 1 and suggests leaving it out.

In addition we noted the following points that relate to specific requirements of EJN.
- The references are not quite in EJN style (too many authors named in in-text citations, all authors should be included in the list and some extraneous information is included for some references e.g. Baxter et al 2000).
- Please include the heading 'Introduction'.
- Author contributions, COI and data statements are needed
- Abbreviation list is needed
- Scale bars needed for Fig 3

When revising the manuscript, please embolden or underline major changes to the text so they are easily identifiable and DO NOT leave 'track change' formatting marks in your paper. Please ensure that you provide a text and a figure file for the Graphical Abstract (as detailed in the instructions below). When carrying out your revisions please refer to the checklist below and visit the EJN author guidelines at www.ejneuroscience.org

When finalized, please upload your complete revised manuscript onto the website, as a Word file (.doc, or .docx). Please also ensure that a complete set of tables and figures is included as separate files, even if these have not changed from the originals. At this stage it is necessary to provide high resolution figures. Please see important instructions below.

Please go into https://mc.manuscriptcentral.com/ejn - Author Centre - manuscripts with decisions where you will find a 'create a revision' link under 'actions'. We ask that you please indicate the way in which you have responded to the points raised by the Editors and Reviewers in a letter. Please upload this response letter as a separate Word (.doc or PDF) file using the file designation "Authors' Response to Reviewers" when uploading your manuscript files. Please DO NOT submit your revised manuscript as a new one. Also, please note that only the Author who submitted the original version of the manuscript should submit a revised version.

If you are able to respond fully to the points raised, we would be pleased to receive a revision of your paper within 12 weeks.

Thank you for your support of this Special Issue of EJN.

Best wishes,

Paul & John

Paul Bolam & John Foxe
co-Editors in Chief, EJN


Reviews:

Reviewer: 1

Comments to the Author
General comments:

(1) This submission investigates the roles of two divisions of the mediodorsal thalamus (MD), the magnocellular (MDmc) and parvocellular (MDpc) divisions, in learning and reward devaluation. This is a valuable area of research because the MD is extensively anatomically connected with the prefrontal cortex (PFC) and there is growing evidence that MD can have a significant influence on PFC neural activity and PFC-dependent functions. Using neurotoxic lesions in macaque monkeys, the authors report that there is a dissociation of function for MDmc and MDpc. Specifically, MDmc supports new learning in the object-in-place task (confirming results in previous publications: Mitchell, Browning, et al., 2007; Browning et al., 2015), whereas MDpc does not; and MDpc contributes to reward devaluation, as can MDmc (in line with published MDmc results). However, this report is based on one monkey with a lesion of MDpc only (subject MDP1), in this case bilateral (as well as one monkey with a bilateral MDmc and MDpc lesion (MDP4), one monkey with a unilateral MDmc and MDpc lesion (MDP2), and one monkey with a MDmc and midline thalamic lesion (MDP3)). Because the consensus in the field appears to be the use of two or more animals for each lesion group to show that an effect generalizes, the single case for each lesion type complicates interpretation of the data.

(2) A couple of suggestions on how to move forward with this submission. One, although the high level of difficulty in primate studies is appreciated, the study would be significantly strengthened with the addition of a second animal with a MDpc lesion only, even though this would take considerable additional time. A second option is to revise the manuscript with a more cautious tone when interpreting data. This might involve reframing the paper as more of a review, with the single-case data prefaced with a phrase such as “the preliminary data show…”. Specific comments below.

Specific comments:

Major:

(3) RE: Object discrimination and reward devaluation, e.g., Table 3. Monkey MDP1 at baseline nearly always chose the M&M option (baseline M:P ratio of 29.5:0.5; as did MDP2). This would seem to bias the “difference score” used to assess devaluation? For example, peanut satiation trials are difficult to interpret because the monkey is already at ceiling, i.e., the monkey cannot choose M&Ms any more often than it had at baseline.

(4) The normative sample for the single case analysis are the control animals in Mitchell, Browning, et al. (2007), three of which (out of four) do not nearly always choose M&Ms over peanuts. Without the ceiling effect (discussed in point 3 above), this would seem to allow larger difference scores for the normative sample? How was this taken into account in the single trial analysis for reward devaluation?

(5) As MDP1 and MDP2 clearly valued M&Ms over peanuts, it might be preferable to separately analyze the M&M satiation trials and peanut satiation trials, rather than combine them as done here for the difference score. That is, what are the statistics on the difference score when using only the M&M satiation trials?

(6) RE: Object-in-place learning, e.g., Table 2 and Figure 4. The animal with a unilateral MD and midline lesion (MDP3) showed recovery of function. For the other MDmc-lesioned animals (MDP2 and MDP4), is performance on posttest1 and posttest 2 significantly different?

(7) One of the authors’ prior studies (Browning et al., 2015) investigated effects of unilateral MDmc lesions on rapid scene learning (prior to additional ablation of orbital and ventral prefrontal cortical regions) and reported that unilateral lesions impair this ability. In the current submission, the possible recovery of function seems like an important observation (one that was briefly noted in the discussion of Browning et al., 2015), and the authors might consider highlighting this in the main text, and possibly “updating” their conclusions on the effect of unilateral MDmc lesions on learning, drawing from available cases with unilateral MDmc lesions.

(8) To date, the authors have performed a number of important studies elucidating the function of MD in learning and reward devaluation. It would be very helpful if these prior results were briefly summarized in the introduction, so that the reader can gain a sense of what is known (and what is not).

Minor:

(9) Is MDP3’s difficulty in learning the associations related to the lesion?

(10) In the discussion, it might be useful to more deeply explore what it means to have attenuated devaluation. It is not clear that the monkeys are doing “less decision making” (line 459); they are making just as many decisions.

(11) RE: MDpc lesion impacting reward devaluation. It is mentioned in the discussion that “MDpc receives non-reciprocal input from the orbitofrontal cortex” (line 522-523). It might be useful to briefly elaborate on this, i.e., how information about reward values might be transmitted from orbitofrontal cortex to MDpc and then to other brain regions. For example, Xaio et al. (2009) have shown that predominantly layer VI (not layer V) of orbitofrontal cortex sends projections to MD. What role could these modulatory (rather than driver) inputs play?


Reviewer: 2

Comments to the Author
The authors reported behavioral data related to learning and reward on four rhesus macaques with different neurotoxic lesions in the MD thalamus. The animals were tested on object-in-place scene discrimination learning task; and object discrimination learning and reinforcement devaluation tasks. The lesions in four animals include respectively MDpc (both sides); unilateral MDpc & contralateral MDmc damage; unilateral MDmc & midline nuclei; and bilateral MDpc & MDmc. The major findings include: bilateral MDpc damage left new learning intact, but damage to MDmc impaired new learning; midline damage disrupted reward association learning; and MDpc damage attenuated devaluation effects after satiety. One of the strengths of the study was that each animal served as its own control.
The experiments were well designed and the findings are interesting for a broad audience of neuroscientists. However, some statements in Introduction and Discussion, especially those about connections between the cortex and MD, need clarification and some references are needed to support other statements. Figure 1 does not seem to be necessary, especially since it focuses on MDpc, the sector affected the least in the tasks employed. In addition, some pathways depicted in Figure 1 are not consistent with the evidence in the literature, and the figure would require major over hall if retained.

Major comments:
1.- Connections of MDpc and prefrontal cortex areas are reciprocal. The authors stated in the introduction and discussion that MDpc receives non-reciprocal inputs from the orbitofrontal cortex, but the evidence in the literature shows that MD sectors and the orbital cortex are reciprocally connected. Some of the references cited by the authors, including Xiao et al 2009, report injections of retrograde tracers in MDpc and MDmc and findings of cortico-thalamic projections from the orbital cortex to MDpc and MDmc. Studies in the literature may report on only one way pathways when retrograde tracers are used, but the pathways are reciprocal. Other studies with retrograde tracer injections in orbital cortex, including areas 12 and 13 also found thalamocortical projections from MDpc and MDmc to the orbital cortex (e. g. Dermon & Barbas 1994, Journal of Comparative Neurology). Injection of bidirectional tracers in the orbital cortex of the monkey has shown anterograde and retrograde labeling in MDmc and MDpc (Cavada et al 2000, Cerebral Cortex; Garcia-Cabezas & Barbas 2017, Cerebral Cortex).
The same can be said about connections between the anterior cingulate cortex (ACC) and MDpc. Xiao et al found corticothalamic projections from ACC areas to MDpc and MDmc, and Dermon & Barbas (1994) found thalamocortical projections from MDmc and MDpc to ACC areas. This evidence is pertinent to the interpretation of the authors’ data.

2.- Orbital areas issue projections to MDpc from layers 5 & 6. The authors stated in Introduction that the driver signal to MDpc “is proposed to originate from the layer 5 cortico-thalamic pathways”. While this may be true, it is important to indicate that cortical projections to MD sectors from prefrontal areas, including the orbital cortex, originate in both layers 5 & 6 (Xiao et al 2009; Garcia-Cabezas & Barbas 2017). In fact, projection neurons from layer 6 predominate, as do projections from many other prefrontal areas to MD. There is no evidence that projections originate exclusively from layer 5 in this system. This also applies for projections from premotor areas directed to the ventral anterior nucleus.

3.- Some references are needed: In the Introduction the authors stated that “The MDpc receives neuromodulatory input from other parts of the midbrain, brainstem and reticular thalamus”. In the Discussion the authors also stated that “Corticothalamic projection neurons to the dorsal thalamus are tenfold more abundant than the thalamocortical inputs to the cortex”. These statements should be backed up with some references from the literature.

4.- Some pathways depicted in Figure 1 are incorrect. The reviewer suggests that this figure be left out, as it focuses on MDpc but not MDmc, an important part of the study. Some of the errors and points for clarification are pointed out below as a way of information:
In Figure 1 projections from MDpc to RTN are depicted as GABAergic, but they are glutamatergic.
Projections from modulatory neurotransmitter systems from the brainstem are depicted in black, while dopaminergic projections from VTA are depicted by a blue arrow. Do the authors want to differentiate VTA dopaminenergic projections to MDpc from other modulatory afferents from the brainstem?
A blue arrow (dopaminergic) connects the dorsal striatum (DS) with the OFC. Such a projection does not exist. The striatum does not contain dopaminergic neurons and does not project monosynaptically to the cortex.
Glutamatergic connections to DS from OFC are depicted with a blue arrow. All the other cortical areas shown in the figure also project to DS.
The FEF is connected with other prefrontal areas, like DLPFC, but in Figure 1 it is not connected at all.
The olfactory tubercle is shown to project to OFC in Figure 1, but the piriform cortex is the major source of olfactory projections to OFC.

Minor corrections:
Line 50, remove the word ‘Although’ (for the sentence to make sense).
Lines 176-177: clarify sentence to show first what happens with a correct response, and then after an incorrect response.
Lines 254-257: Explain clearly the devaluation issue; it is confusing as briefly stated.
Line 584: please change ‘laminar’ to: laminae (or layers)
Line 574: “…to disrupt widespread disrupted neural..” please remove the word ‘disrupted’
Lines 596-601; break up the long sentence for clarity.
Line 318: Spell out NMDA.
Line 325: Check square typo (symbol?).
Line 391: verses should be versus (this is also misspelled elsewhere).
Please, mention in the methods the side (left/right) of the lesioned hemi thalami.

_____________________________________________________________________________________

Authors’ Response	05.05.2018

_____________________________________________________________________________________

Thank you very much to both Reviewers for their comments – they are both very thorough and have provided thoughtful and helpful suggestions. The changes we have made are all highlighted in the manuscript with red text.

Reviewer: 1

Comments to the Author
General comments:

(1) This submission investigates the roles of two divisions of the mediodorsal thalamus (MD), the magnocellular (MDmc) and parvocellular (MDpc) divisions, in learning and reward devaluation. This is a valuable area of research because the MD is extensively anatomically connected with the prefrontal cortex (PFC) and there is growing evidence that MD can have a significant influence on PFC neural activity and PFC-dependent functions. Using neurotoxic lesions in macaque monkeys, the authors report that there is a dissociation of function for MDmc and MDpc. Specifically, MDmc supports new learning in the object-in-place task (confirming results in previous publications: Mitchell, Browning, et al., 2007; Browning et al., 2015), whereas MDpc does not; and MDpc contributes to reward devaluation, as can MDmc (in line with published MDmc results). However, this report is based on one monkey with a lesion of MDpc only (subject MDP1), in this case bilateral (as well as one monkey with a bilateral MDmc and MDpc lesion (MDP4), one monkey with a unilateral MDmc and MDpc lesion (MDP2), and one monkey with a MDmc and midline thalamic lesion (MDP3)). Because the consensus in the field appears to be the use of two or more animals for each lesion group to show that an effect generalizes, the single case for each lesion type complicates interpretation of the data.

(2) A couple of suggestions on how to move forward with this submission. One, although the high level of difficulty in primate studies is appreciated, the study would be significantly strengthened with the addition of a second animal with a MDpc lesion only, even though this would take considerable additional time. A second option is to revise the manuscript with a more cautious tone when interpreting data. This might involve reframing the paper as more of a review, with the single-case data prefaced with a phrase such as “the preliminary data show…”.  Specific comments below.

We have worked with this reviewer’s second option and have revised the manuscript, in particular we have re-written the discussion to highlight that these are preliminary data because we have single case monkeys each with a different MDpc and/or MDmc lesion.

Specific comments:

Major:

(3) RE: Object discrimination and reward devaluation, e.g., Table 3. Monkey MDP1 at baseline nearly always chose the M&M option (baseline M:P ratio of 29.5:0.5; as did MDP2). This would seem to bias the “difference score” used to assess devaluation? For example, peanut satiation trials are difficult to interpret because the monkey is already at ceiling, i.e., the monkey cannot choose M&Ms any more often than it had at baseline.

To address this we have included a further analysis of the proportion of adaptive responses made by each monkey during just the devaluation tests. This analysis, unlike the difference score, is independent of the baseline data. This is included on page 18 and we have included a related Figure 5A and 5B.

(4) The normative sample for the single case analysis are the control animals in Mitchell, Browning, et al. (2007), three of which (out of four) do not nearly always choose M&Ms over peanuts. Without the ceiling effect (discussed in point 3 above), this would seem to allow larger difference scores for the normative sample? How was this taken into account in the single trial analysis for reward devaluation?

As per point 3 above, we have included a further analysis of the proportion of adaptive responses for each monkey and this is based on the devaluation test and does not include baseline data.

(5) As MDP1 and MDP2 clearly valued M&Ms over peanuts, it might be preferable to separately analyze the M&M satiation trials and peanut satiation trials, rather than combine them as done here for the difference score. That is, what are the statistics on the difference score when using only the M&M satiation trials?

We have computed these difference scores from Table 3 of the manuscript and include them here below for the M&M satiation trials. We have not included them in the text as it is not the standard way to conduct these analyses given that in total four devaluation tests with satiation of food rewards were conducted in the following order (1: 1 with peanuts, 2: 1 with M&Ms, 3: 1 with peanuts and then 4: 1 with M&Ms).

| **Monkeys** | **Food devaluation Test 1** | | **Food devaluation Test 2** | | **Overall Mean** | |
| --- | --- | --- | --- | --- | --- | --- |
|  | **Diff score** | **Single case analysis** | **Diff score** | **Single case analysis** | **Diff Score** | **Single case analysis** |
| MDP1-Bi MDpc | -0.5 | 0.088 | 14.5 | 0.854 | 14.0 | 0.824 |
| MDP2-Uni MDpc x Uni MDmc | 10.5 | 0.726 | 13.5 | 0.943 | 12.0 | 0.893 |
| MDP3-Uni MDmc + midline | 9.0 | 0.549 | 13.5 | 0.943 | 12.25 | 0.929 |
| MDP4-Bi MDpc+Bi MDmc | 1.0 | 0.114 | 0.5 | 0.227 | 1.5 | 0.117 |

The difference scores related to the number of M&M rewarded objects chosen by each monkey during the two devaluation tests. Individual difference scores for Test 1 and Test 2 are compared to the normative sample of unoperated controls from Mitchell et al., (2007): Mean Test 1 = 12.5, S.D = 4.56 and N = 4; Mean Test 2 = 12.87, S.D = 7.30 and N = 4; Overall Mean = 12.5, S.D = 4.66 and N = 4).

(6) RE: Object-in-place learning, e.g., Table 2 and Figure 4. The animal with a unilateral MD and midline lesion (MDP3) showed recovery of function. For the other MDmc-lesioned animals (MDP2 and MDP4), is performance on posttest1 and posttest 2 significantly different?

The statistical analysis revealed that the performance on Posttest1 versus Posttest2 is not statistically significant for MDP2, p = 0.009; for MDP4 the difference was significant, p = 0.133. This information has been included in the results section on pages 14 and 15.

(7) One of the authors’ prior studies (Browning et al., 2015) investigated effects of unilateral MDmc lesions on rapid scene learning (prior to additional ablation of orbital and ventral prefrontal cortical regions) and reported that unilateral lesions impair this ability. In the current submission, the possible recovery of function seems like an important observation (one that was briefly noted in the discussion of Browning et al., 2015), and the authors might consider highlighting this in the main text, and possibly “updating” their conclusions on the effect of unilateral MDmc lesions on learning, drawing from available cases with unilateral MDmc lesions.

We have done this now and these changes have been included on page 22.

(8) To date, the authors have performed a number of important studies elucidating the function of MD in learning and reward devaluation. It would be very helpful if these prior results were briefly summarized in the introduction, so that the reader can gain a sense of what is known (and what is not).

These have been summarised in the introduction on page 3.

Minor:

 (9) Is MDP3’s difficulty in learning the associations related to the lesion?

We don’t believe that it is and have indicated this in the text on page 19.

(10) In the discussion, it might be useful to more deeply explore what it means to have attenuated devaluation. It is not clear that the monkeys are doing “less decision making” (line 459); they are making just as many decisions.

Additional points have been made in the discussion on page 19-20.

(11) RE: MDpc lesion impacting reward devaluation. It is mentioned in the discussion that “MDpc receives non-reciprocal input from the orbitofrontal cortex” (line 522-523). It might be useful to briefly elaborate on this, i.e., how information about reward values might be transmitted from orbitofrontal cortex to MDpc and then to other brain regions. For example, Xaio et al. (2009) have shown that predominantly layer VI (not layer V) of orbitofrontal cortex sends projections to MD. What role could these modulatory (rather than driver) inputs play?

We have provided a further, brief explanation on page 20.

Reviewer: 2

Comments to the Author
The authors reported behavioral data related to learning and reward on four rhesus macaques with different neurotoxic lesions in the MD thalamus. The animals were tested on object-in-place scene discrimination learning task; and object discrimination learning and reinforcement devaluation tasks. The lesions in four animals include respectively MDpc (both sides); unilateral MDpc & contralateral MDmc damage; unilateral MDmc & midline nuclei; and bilateral MDpc & MDmc. The major findings include: bilateral MDpc damage left new learning intact, but damage to MDmc impaired new learning; midline damage disrupted reward association learning; and MDpc damage attenuated devaluation effects after satiety. One of the strengths of the study was that each animal served as its own control.
The experiments were well designed and the findings are interesting for a broad audience of neuroscientists. However, some statements in Introduction and Discussion, especially those about connections between the cortex and MD, need clarification and some references are needed to support other statements. Figure 1 does not seem to be necessary, especially since it focuses on MDpc, the sector affected the least in the tasks employed. In addition, some pathways depicted in Figure 1 are not consistent with the evidence in the literature, and the figure would require major over hall if retained.
We have deleted Figure 1.

Major comments:
1.- Connections of MDpc and prefrontal cortex areas are reciprocal. The authors stated in the introduction and discussion that MDpc receives non-reciprocal inputs from the orbitofrontal cortex, but the evidence in the literature shows that MD sectors and the orbital cortex are reciprocally connected. Some of the references cited by the authors, including Xiao et al 2009, report injections of retrograde tracers in MDpc and MDmc and findings of cortico-thalamic projections from the orbital cortex to MDpc and MDmc. Studies in the literature may report on only one way pathways when retrograde tracers are used, but the pathways are reciprocal. Other studies with retrograde tracer injections in orbital cortex, including areas 12 and 13 also found thalamocortical projections from MDpc and MDmc to the orbital cortex (e. g. Dermon & Barbas 1994, Journal of Comparative Neurology). Injection of bidirectional tracers in the orbital cortex of the monkey has shown anterograde and retrograde labeling in MDmc and MDpc (Cavada et al 2000, Cerebral Cortex; Garcia-Cabezas & Barbas 2017, Cerebral Cortex). 
The same can be said about connections between the anterior cingulate cortex (ACC) and MDpc. Xiao et al found corticothalamic projections from ACC areas to MDpc and MDmc, and Dermon & Barbas (1994) found thalamocortical projections from MDmc and MDpc to ACC areas. This evidence is pertinent to the interpretation of the authors’ data.

The neuroanatomy section of the introduction has been updated on pages 3-4 and implications of the specific neuroanatomy have been discussed in relation to both tasks on pages 20-24 of the discussion.

2.- Orbital areas issue projections to MDpc from layers 5 & 6. The authors stated in Introduction that the driver signal to MDpc “is proposed to originate from the layer 5 cortico-thalamic pathways”. While this may be true, it is important to indicate that cortical projections to MD sectors from prefrontal areas, including the orbital cortex, originate in both layers 5 & 6 (Xiao et al 2009; Garcia-Cabezas & Barbas 2017). In fact, projection neurons from layer 6 predominate, as do projections from many other prefrontal areas to MD. There is no evidence that projections originate exclusively from layer 5 in this system. This also applies for projections from premotor areas directed to the ventral anterior nucleus. 

We have reworded this in our introduction and discussion to make it clearer that both layers 5 and 6 innervate the thalamus and never layer 5 alone.

3.- Some references are needed: In the Introduction the authors stated that “The MDpc receives neuromodulatory input from other parts of the midbrain, brainstem and reticular thalamus”. In the Discussion the authors also stated that “Corticothalamic projection neurons to the dorsal thalamus are tenfold more abundant than the thalamocortical inputs to the cortex” (Jones, E.G. *The Thalamus* (Cambridge University Press, Cambridge, 2007).These statements should be backed up with some references from the literature.

We have included the reference of Jones (2007) for the second reference, and indicate the review of Mitchell (2015) provides additional details about the inputs from midbrain, brainstem and reticular thalamus.

4.- Some pathways depicted in Figure 1 are incorrect. The reviewer suggests that this figure be left out, as it focuses on MDpc but not MDmc, an important part of the study. Some of the errors and points for clarification are pointed out below as a way of information: 
In Figure 1 projections from MDpc to RTN are depicted as GABAergic, but they are glutamatergic.
Projections from modulatory neurotransmitter systems from the brainstem are depicted in black, while dopaminergic projections from VTA are depicted by a blue arrow. Do the authors want to differentiate VTA dopaminenergic projections to MDpc from other modulatory afferents from the brainstem?
A blue arrow (dopaminergic) connects the dorsal striatum (DS) with the OFC. Such a projection does not exist. The striatum does not contain dopaminergic neurons and does not project monosynaptically to the cortex.
Glutamatergic connections to DS from OFC are depicted with a blue arrow. All the other cortical areas shown in the figure also project to DS.
The FEF is connected with other prefrontal areas, like DLPFC, but in Figure 1 it is not connected at all.
The olfactory tubercle is shown to project to OFC in Figure 1, but the piriform cortex is the major source of olfactory projections to OFC.

We have deleted this Figure.

Minor corrections:
Line 50, remove the word ‘Although’ (for the sentence to make sense). Deleted
Lines 176-177: clarify sentence to show first what happens with a correct response, and then after an incorrect response. Done.
Lines 254-257: Explain clearly the devaluation issue; it is confusing as briefly stated. Done on page 10.
Line 584: please change ‘laminar’ to: laminae (or layers) Done
Line 574: “…to disrupt widespread disrupted neural..” please remove the word ‘disrupted’ Done
Lines 596-601; break up the long sentence for clarity.  Done, now on page 21 – lines 609-615.        
Line 318: Spell out NMDA. Done
Line 325: Check square typo (symbol?).
Line 391: verses should be versus (this is also misspelled elsewhere). Done
Please, mention in the methods the side (left/right) of the lesioned hemi thalami. Done

_____________________________________________________________________________________

2nd Editorial Decision	06.06.2018

_____________________________________________________________________________________

Macaque parvocellular mediodorsal thalamus: dissociable contributions to learning and adaptive decision-making

Dear Anna,

Your revised manuscript has been re-evaluated by the Editorial team and we are pleased to inform you that it will be accepted for publication in Special Issue of EJN following a few tiny revisions.  Please supply:
1) Data statement
2) Competing interests statement
3) Graphical abstract

Once done, we will send it off to the publishers.  

When finalised, please upload your complete revised manuscript onto the website as a Word (.doc or .docx), or .rtf file.  Please also ensure that a complete set of tables and figures is included as separate files, even if these have not changed from the originals.  At this stage it is necessary to provide high resolution figures.  Please see important instructions below.

Please go into https://mc.manuscriptcentral.com/ejn - Author Centre - manuscripts with decisions where you will find a 'create a revision' link under 'actions'. We ask that you please indicate the way in which you have responded to the points raised by the Editors and Reviewers in a letter. Please upload this response letter as a separate Word (.doc) file using the file designation "Authors' Response to Reviewers" when uploading your manuscript files. Please DO NOT submit your revised manuscript as a new one.  Also, please note that only the  Author who submitted the original version of the manuscript should submit a revised version.

If you are able to respond fully to the points raised, we shall be pleased to receive a revision of your paper within 30 days.

Thank you for your support of this Special Issue of EJN in honour of our old friend.

Best wishes,

Paul & John
co-Editors in Chief, EJN

_____________________________________________________________________________________

Authors’ Response	03.07.2018

_____________________________________________________________________________________

Dear Paul and John

This is wonderful news! We are very pleased and honoured to be able to be a part of this Special Issue in honour of Ray.

We have included a graphical abstract, data statement and conflict of interest statement to the manuscript.

I trust all is now in order.

With best wishes
Anna
